# Use of Cross-Taxon Congruence for Hotspot Identification at a Regional Scale

**DOI:** 10.1371/journal.pone.0040018

**Published:** 2012-06-26

**Authors:** Simone Fattorini, Roger L. H. Dennis, Laurence M. Cook

**Affiliations:** 1 Water Ecology Team, Department of Biotechnology and Biosciences, University of Milano Bicocca, Milan, Italy; 2 Azorean Biodiversity Group, Departamento de Ciências Agrárias CITA-A, Universidade dos Açores, Pico da Urze, Angra do Heroísmo, Portugal; 3 Department of Biological and Medical Sciences, Oxford Brookes University, Headington, Oxford, United Kingdom; 4 Institute for Environment, Sustainability and Regeneration, Staffordshire University, Stoke on Trent, United Kingdom; 5 Faculty of Life Sciences, The University of Manchester, Manchester, United Kingdom; University of Western Australia, Australia

## Abstract

One of the most debated problems in conservation biology is the use of indicator (surrogate) taxa to predict spatial patterns in other taxa. Cross-taxon congruence in species richness patterns is of paramount importance at regional scales to disclose areas of high conservation value that are significant in a broader biogeographical context but yet placed in the finer, more practical, political context of decision making. We analysed spatial patterns of diversity in six arthropod taxa from the Turkish fauna as a regional case study relevant to global conservation of the Mediterranean basin. Although we found high congruence in cross-taxon comparisons of species richness (0.241<*r*<0.645), hotspots of different groups show limited overlap, generally less than 50 per cent. The ability of a given taxon to capture diversity of other taxa was usually modest (on average, 50 percent of diversity of non-target taxa), limiting the use of hotspots for effective conservation of non-target groups. Nevertheless, our study demonstrates that a given group may partially stand in for another with similar ecological needs and biogeographical histories. We therefore advocate the use of multiple sets of taxa, chosen so as to be representative of animals with different ecological needs and biogeographical histories.

## Introduction

A number of studies have tested biodiversity hotspot coincidence, i.e. whether the geographical patterns of species richness in one taxon act as a surrogate for those in other taxa [1]–[6]. Typically, studies over broad regions have found high cross-taxon congruence in species richness patterns [5]–[13], although there are significant exceptions [14], [15] and the causal mechanisms underlying variation in the strength of cross-taxon correlation across taxonomic groups, spatial scale and ecosystem types remain elusive [16]. At very low resolutions, cross-taxon congruence in species diversity values and locations of hotspots can be expected because of common responses of different organisms to large-scale variations in climate and geologic history [17], [18], and as a consequence of statistical differences in range size [5].

Although useful to elucidate global patterns of biodiversity, these studies are less important from a practical point of view, because most conservation undertakings are carried out at regional scales within state boundaries. Hotspot identification at a regional scale discloses areas of high interest for conservation investment [4], [19]. Thus, it is of paramount importance to know if there is cross-taxon congruence, and hence if certain taxa can be used as surrogates for others, at a regional scale. Yet, cross-taxon covariation at regional scales has not been explored. In this article, we analyse cross-taxon congruence to assess its value as a tool at a regional scale.

**Figure 1:**
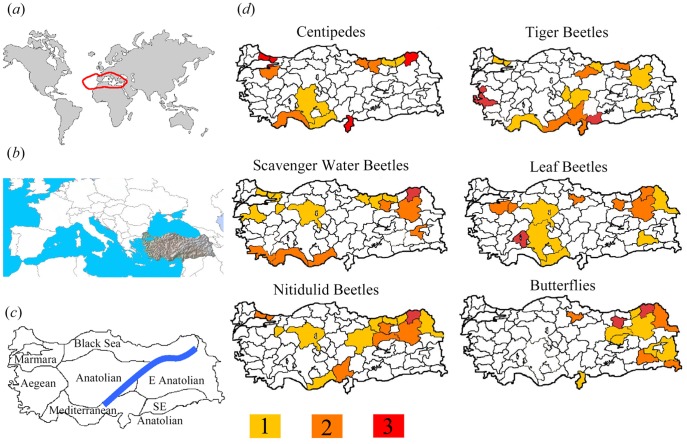
The Mediterranean global hotspot (a), location of the study area (Turkey) (b), its main biogeographical regions (c), and hotspots for different arthropod groups (d). Position of the Anatolian Diagonal, a major biogeographical barrier, is shown in panel c. For each group, hotspots were calculated as the first 10 per cent rank in three different diversity metrics (species richness, species richness-area ratio, residual from the species-area relationship). Different grey tones indicate if a certain hotspot has been identified by one, two or all three metrics.

For this purpose we selected six arthropod taxa (centipedes, tiger beetles, water scavenger beetles, nitidulid beetles, leaf beetles, and butterflies) with different ecological needs (carnivores and herbivores) from the Turkish fauna (Fig. 1). The Mediterranean basin is one of the global hotspots under serious threat [8], [20], [21] and Turkey is one of the foremost centres of Mediterranean and European biodiversity [22]–[25]; thus preservation of Turkish wilderness is of both local and global importance.

For the analysis hotspots are considered to be the richest 10 per cent of the units surveyed, in this case the top 7 of 67 administrative areas. The most direct metric to use would be species richness per area. Because the areas are of different sizes, however, we also used two other diversity metrics: species/area ratios [8], [20] and the differences between observed values of species richness and values predicted from the species-area relationship (residuals from SAR) [10]. We then ask the following questions:

1. Do different taxa show congruent variation in diversity values across areas within a region (*cross-taxon congruence*)?
2. Do the top ranking areas actually contain a large fraction of the species that comprise the group (*within-taxon conservation effectiveness*)?
3. Are hotspots of a given taxon able to capture a high fraction of the diversity of other taxa (*cross-taxon conservation effectiveness*)?

The first question is generally addressed by correlating values of diversity between different taxa [26], [27]. However, statistically positive congruence in cross-taxon correlations does not necessarily imply identical selection of areas as putative hotspots [28]. For example, a statistical significance could emerge from high congruence in the order of areas with *low* values of diversity metrics (i.e. richness, residuals, or species/area ratios), while we are most interested in searching for congruence among the *highest* values. Thus, it is important not simply to assess cross-taxon correlation in diversity metrics, but also the extent to which the different taxa agree in their identification of hotspots. The second question arises from the fact that it is always possible to select as hotspots the areas that maximize a given diversity metric, but it is also important that these metrics provide satisfactory estimates of species richness [29]. The third question relates to the current practice of using indicator taxa to predict spatial patterns in other taxa [2]–[4], [30]–[33].

## Methods

### Taxa Analysed and Geographic Coding

The study is entirely based on published species records. We gathered data for the following taxa: centipedes (Chilopoda), tiger beetles (Coleoptera Cicindelidae), scavenger water beetles (Coleoptera Hydrophilidae, gen. *Laccobius*), nitidulid beetles (Coleoptera Nitidulidae), leaf beetles (Coleoptera Chrysomelidae Cryptocephalinae), and butterflies (Lepidoptera Papilionoidea) (see [34] for details). We coded records of species and subspecies for all former 71 administrative areas (provinces) because some regional data are reported in the literature with reference to these areas (lists of references and species distribution data are provided as electronic Supporting Information S1). Areas which have been consistently undersampled were omitted [34]. The final number of areas considered for analyses was 67. These areas have a relatively low variation in their size (mean value ± SD: 11265.150±6910.143 km^2^). When information was available, we have considered both species and subspecies. The current taxonomic dividing line between species and subspecies, as applied to most Turkish arthropods, is arguably arbitrary. Subspecies, as well as species, are regarded as representing ‘evolutionary significant units’ [35]. In this there is application of the ‘phylogenetic species concept’ as the smallest biological entities that are diagnosable and/or monophyletic [36].

### Cross-taxon Congruence

Cross-taxon species-richness correlations were tested using Pearson coefficients (*r*). To remove the possible area effect on the relationships between species richness of different taxa we have used two approaches. The simplest was to calculate the species/area ratio. However, to account for the possible non-linearity in the species-area relationship (SAR), and different responses among groups, we modelled for each group a SAR with the Arrhenius power function [37]–[40] and used residuals as an area corrected measure of diversity [10]. To control for spatial non-independence, statistical significance of correlation coefficients was calculated under an estimated effective sample size given the observed degree of spatial autocorrelation [5] using Dutilleul’s algorithm [41], [42].

For hotspot identification, we ranked areas according to residuals and considered for each group the first 10 per cent of areas [28], [39], [43].

To assess how well the different taxa agree in their identification of hotspots and to evaluate the performance of each taxon separately by determining whether it could identify hotspots previously identified by the other taxa, we calculated the overlap (percent similarity) of identified hotspots among taxa. For each of the three measures of diversity we ranked areas and selected as hotspots the highest 10 per cent of ranks from all taxa. For a given measure, the percent similarity among the six taxa was determined as the number of hotspots shared by each pair of taxa within the first seven areas. Then we assessed the probability of obtaining the same number of shared hotspots by chance alone. To calculate the probability of obtaining the same number of shared hotpots, the ratios between two binomial coefficients were obtained using the following formula:
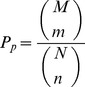
where *N*  =  number of areas (67 in all cases), *n*  =  number of areas identified as hotspots (7 in all cases), *M*  =  *N-p*, *m*  =  *n-p*, with *p*  =  number of shared hotspots.

To explore congruence among different methods in identifying hotspots for a given taxon, we calculated for each taxon the percent similarity of identified hotspots among methods and the pairwise correlations among them.

### Conservation Effectiveness

To measure the effectiveness of selected hotspots to capture species richness within taxa, we calculated for each type of hotspot the fraction of included species [29], [37]. In cross-taxon analyses, to measure the performance of priority sets based on indicator groups, we calculated the fraction of non-target species captured by hotspots of indicator taxa [4].

To assess if the total areas included in the hotspots varied according to the method used to identify the hotspots and the animal group considered, a main effects ANOVA was applied using hotspot surface as a dependent variable (log-transformed to achieve normality) and taxon and criteria (richness, species/area ratio, residuals from species-area relationship) as categorical factors. Criteria had a significant effect (*P*<0.000001), whereas taxa had no significant effect (*P* = 0.378). Fisher LSD tests for post-hoc comparisons were therefore used to investigate differences between the three criteria.

Many tests were made on the same data set, thus increasing the risk of significant results arising owing to chance alone. We believe, however, that decreasing the significance levels would result in an even higher risk of ignoring true relationships. Therefore, as in other studies dealing with cross-taxon analysis (e.g. [30]), and in accordance with the suggestions of Moran [44], we did not apply the Bonferroni correction, but focused on *P*-values and consistency of results.

## Results and Discussion

### Cross-taxon Congruence

Patterns of cross-taxon species richness were significantly and positively correlated in all groups (0.241<*r*<0.645; 0.0001<*P*<0.05; *N* = 67). The use of species/area ratios (0.251<*r*<0.814; 0.0001<*P<*0.05; *N* = 67) gave significantly positive correlations in all cases. Residuals from the SAR gave significantly positive correlations in all cases (0.312<*r*<0.546; 0.0001<*P*<0.05; *N* = 67), except for the residuals of leaf and tiger beetles (*r* = 0.153; *P* = 0.26), residuals of centipedes and butterflies (*r* = 0.150; *P* = 0.25), and possibly residuals of butterflies and tiger beetles (*r* = 0.262; *P* = 0.055) and butterflies and scavenger beetles (*r* = 0.236; *P* = 0.052).

Thus, pairwise correlations varied according to the method and the taxon considered. Also, no area was identified as a hotspot for all taxa (Fig. 1d), although one province (Artvin) was recognized as a hotspot by species/area ratios and residuals for all groups except tiger beetles, and the use of richness recovered two provinces (İçel and Erzurum) as hotspots for all taxa except one association (centipedes and butterflies, respectively). Thus, although different metrics of diversity were statistically correlated among groups, this does not guarantee congruence among hotspots. Statistical significance was likely due to high congruence in the order of areas with *low* values of the three metrics, while there was poor overall congruence among the *highest* values. As a matter of fact, in all three diversity metrics, between group correlations show a strong relationship at low diversity, whereas there is more scatter at higher diversity values (see Supporting Information S2). Thus, overall cross-taxon congruence does not show with any certainty that different groups have similar spatial distributions and hence similar hotspots. Although overall rankings were significantly correlated in all groups, the overlap of identified hotspots was generally less than 50 per cent (richness: mean ± SD: 38.10±14.95, range: 14–57; residuals from the SAR: mean ± SD: 25.71±15.46, range: 0–43; species/area ratios: mean ± SD: 41.90±18.28, range: 14–71; *N* = 15 in all cases), thus indicating that overall congruence was mostly due to non-hotspot areas.

Hotspots tend to be scattered throughout the biogeographical regions of Turkey. However, we found several instances of per cent overlap higher than 50 per cent (*P*<5×10^−5^) in pairwise comparisons, and, for certain groups the spatial distribution of hotspots did identify particular biogeographical regions.

Hotspots for centipedes (typically associated with soil litter of forest biotopes), tiger beetles (which include several montane species) and scavenger water beetles (associated with freshwater biotopes) were concentrated in the areas along the northern and southern chains (Black Sea, Mediterranean and SE Anatolian regions) (Fig. 1c,d), characterised by high rainfall and dense forest.

It is also interesting that the geographic distribution of hotspots of nitidulid beetles and butterflies is concentrated mostly east of the ‘Anatolian Diagonal’, a mountain range which extends from the northeast towards the southwest, and which represents an important biogeographical discontinuity [24] (Fig. 1c). Finally, the distribution of hotspots of leaf beetles (which are mostly associated with Mediterranean forests) fits well with the distribution of the Mediterranean forest vegetation in Turkey [45].

### Cross-method Congruence

Because we used three different metrics of diversity, we also explored their congruence. Pairwise correlations between values of species richness, species-area ratios and SAR residuals were significantly positive in all taxa (always 0.235<*r<*0.969, *P*<0.001, *N* = 67).

However, for each taxon, the overlap of identified hotspots was never more than 45 per cent among the three methods, although the pairwise comparisons of methods revealed many instances of overlap>45 per cent.

### Within-taxon Conservation Effectiveness

Hotspots included about 68–80 per cent of total richness of each group when identified using species richness (mean ± SD: 74.25±4.36) and SAR residuals (73.34±3.42), and about 48–73 per cent (61.55±8.50) if identified using the species/area ratios. However, the total area comprised in each set of hotspots varied greatly among methods. Hotspots localised using species richness and SAR residuals included more species, but also a larger total area, than those obtained using species/area ratios (LSD tests, *P*<0.0001 in all comparisons; no difference was found between taxa). As a rule, the use of species richness hotspots or hotspots from SAR residuals instead of species/area ratio hotspots would determine very moderate increases (usually less than 20 per cent) in included species, but with enormous increases in the included area (165 to 239 per cent for richness based hotspots, and 24 to 121 per cent for residuals based hotspots). This is important if the conservation objective is to maximise the number of species within the smallest area.

### Cross-taxon Conservation Effectiveness

Although overlap of hotspots based on different indicator groups was only moderate, representation of non-target taxa was nonetheless good (table 1). Different indicator taxa were able to capture, on average, about 50 percent of diversity of non-target taxa (49 to 66 per cent for species richness, 44 to 52 for the species/area ratios, and 45 to 62 per cent for the residuals from the SARs). For most groups, the species diversity captured by indicator taxa was lower than that captured by hotspots of the group concerned. However, in some cases, indicator taxa performed equally or better than the taxon of concern itself. This indicates that, although hotspots do not overlap consistently, those selected for a given taxon nevertheless capture relatively large fractions of diversity in non-target taxa.

**Table 1:** Percentage of species richness of target taxa captured by hotspots of indicator taxa (in italics, percentages of species of each group included in the hotspots identified by the group itself).

Hotspots defined according to species richness						
	Indicator taxon					
Target taxon	Centipedes	Tiger beetles	Water scavenger beetles	Leafbeetles	Nitidulidbeetles	Butterflies
Centipedes	*70.97*	40.32	54.03	48.39	45.97	36.29
Tiger beetles	62.50	*72.50*	62.50	62.50	67.50	37.50
Water scavenger beetles	64.00	60. 0	*80.00*	72.00	80.00	72.00
Leaf beetles	56.99	43.01	67.74	*77.42*	62.37	56. 9
Nitidulid beetles	61.29	43.23	63.23	56.13	*68.39*	56.13
Butterflies	71.31	58.67	77.30	77.09	75.38	*76.23*

**Hotspots defined according to species/area ratios**						
	**Indicator taxon**					
**Target taxon**	**Centipedes**	**Tiger beetles**	**Water scavenger beetles**	**Leaf** **beetles**	**Nitidulid** **beetles**	**Butterflies**
Centipedes	*59.68*	53.23	37.90	50.00	50.00	47.58
Tiger beetles	55.00	*67.50*	32.50	37.50	42.50	45.00
Water scavenger beetles	52.00	40.00	*48.00*	48.00	52.00	52.00
Leaf beetles	34.41	30.11	44.09	*60.22*	43.01	41.94
Nitidulid beetles	54.19	39.36	48.39	47.10	*60.65*	50.32
Butterflies	62.10	61.88	56.32	72.38	59.53	*73.23*

**Hotspots defined according to residuals from the species area relationship**						
	**Indicator taxon**					
**Target taxon**	**Centipedes**	**Tiger beetles**	**Water scavenger beetles**	**Leaf** **beetles**	**Nitidulid** **beetles**	**Butterflies**
Centipedes	*72.58*	41.94	51.61	50.00	54.84	38.71
Tiger beetles	60.00	*67.50*	57.50	37.50	72.50	40.00
Water scavenger beetles	56.00	60.00	*76.00*	64.00	72.00	52.00
Leaf beetles	47.31	40.86	61.29	*72.04*	55.91	48.39
Nitidulid beetles	55.48	47.74	61.94	58.07	*76.77*	47.10
Butterflies	68.09	61.88	76.02	68.95	74.73	*75.16*

In some cases, indicator taxa performed equally or better than the taxon of concern itself. Butterflies performed poorly in capturing diversity of other groups, whereas other groups usually captured high proportion of butterfly diversity.

No single taxon performed consistently best in capturing diversity for other taxa for all three methods (species richness, species/area ratios and SAR residuals), although scavenger beetles, centipedes and nitidulid beetles were good surrogates for other taxa in many circumstances. Butterflies, which are commonly considered an ‘umbrella group’ [46] perform poorly in capturing diversity of other groups, whereas other groups usually capture high proportions of butterfly diversity.

A common problem with all methods applied here is that they are strongly influenced by widespread species, which are of lower conservation value than range-restricted or endemic species. In our analyses we obtained, for some groups, the unexpected result that hotspots of indicator taxa captured similar numbers of species as hotspots did of the target taxon (or, paradoxically, sometimes even more). If areas that ranked highest in species number (or derived metrics) show large overlap in species composition there could be a highly nested pattern, so that adding other rich areas does not necessarily increase species number. Since the most widespread species are those most likely to recur they tend to have a diminishing effect on diversity. Use of algorithms of complementarity [3], [47], [48] only partially circumvents this problem. In the attempt to include as many species as possible for different groups, areas which contributed few so-far-unrepresented species could be omitted, but if endemics are localised in such poor areas, there is a substantial risk of losing them from the final set. The finding that groups that contain many localised species are less well indicated by other taxa [4] may be a reflection of this problem.

### Conclusions

In cross-taxon comparisons our results showed high congruence. However, this covariation is a consequence of concordance between the lowest values, and to that extent, does not provide a good indicator of hotspot distribution. Our study also showed that cross-taxon congruence does not imply that geographical patterns of richness in one group act as a surrogate for those in other groups. The ability of a given taxon to capture diversity of other taxa was usually moderate, thus questioning the use of hotspots for effective conservation of non-target groups.

Although generalised surrogacy is unlikely, our study nevertheless demonstrates that a given group may partially stand in for another with similar ecological needs and biogeographical histories. Thus, we do not propose to dismiss the use of indicator taxa, but when using them we advocate the use multiple sets of taxa, chosen so as to be representative of animals with different ecological needs and biogeographical histories.

## Supporting Information

Supporting Information S1 Distribution of Centipedes, Tiger beetles, Water scavenger beetles, Leaf beetles, Nitidulid beetles, and Butterflies in Turkey.
(XLS)

Supporting Information S2 Cross-taxon correlations for species richness (a), species/area ratio (b), and residuals from the species-area relationship (c).
(PDF)
