# Supplementary figures and images for: Use of Cross-Taxon Congruence for Hotspot Identification at a Regional Scale
Source: PLoS One. 2012 Jun 26;7(6):e40018. doi: 10.1371/journal.pone.0040018 (PMC3383703; doi:10.1371/journal.pone.0040018)

(a)

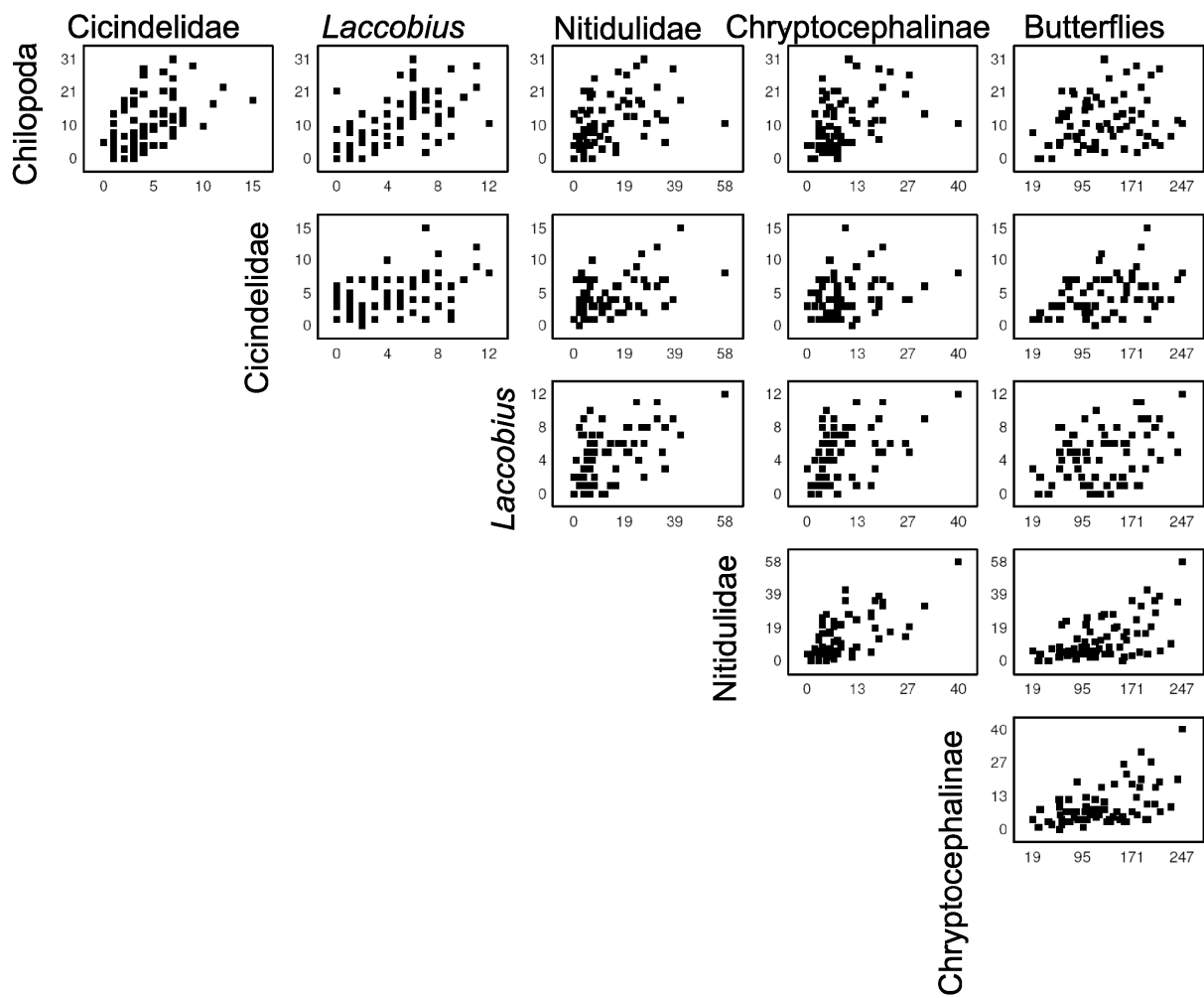

(b)

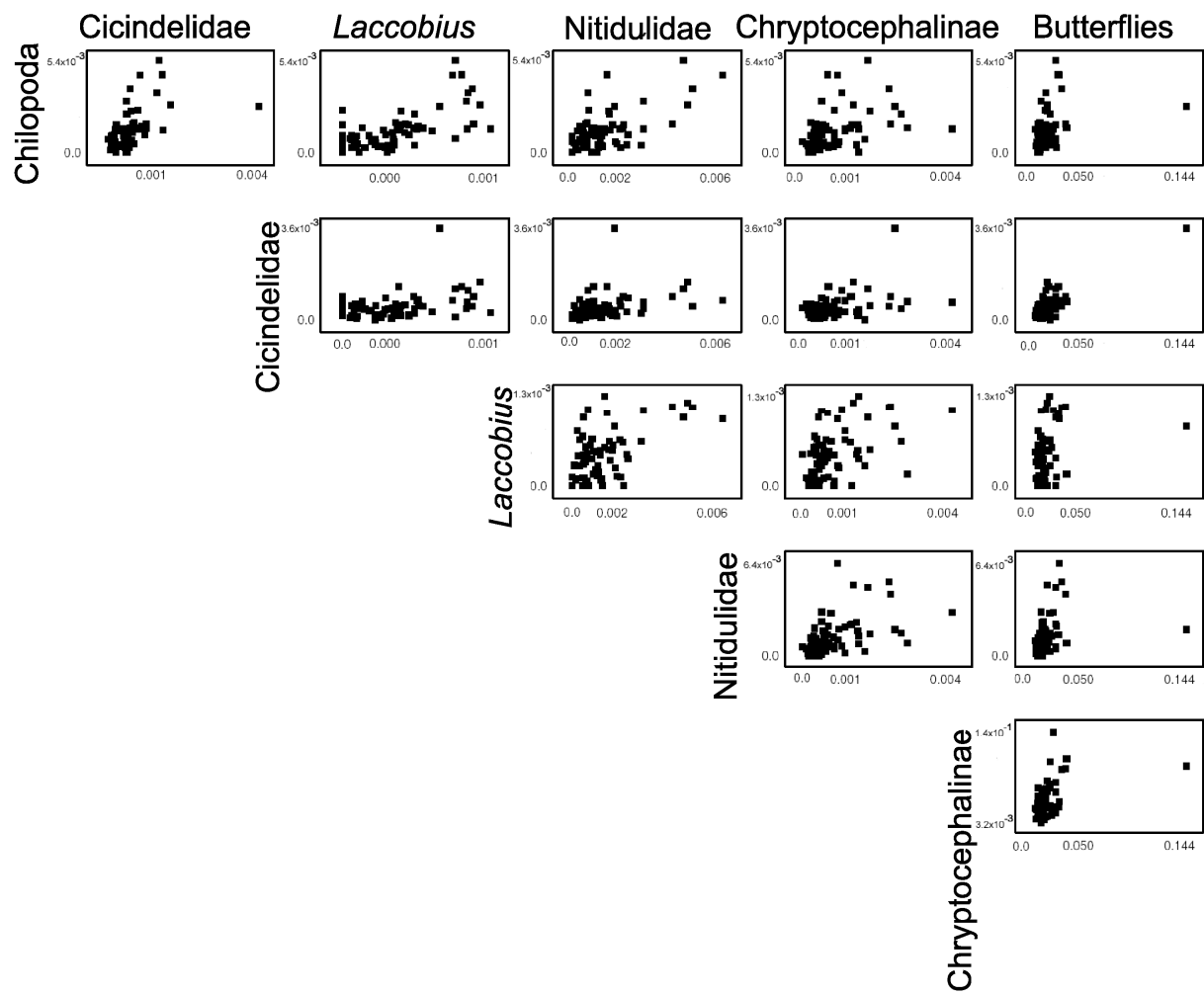

(c)

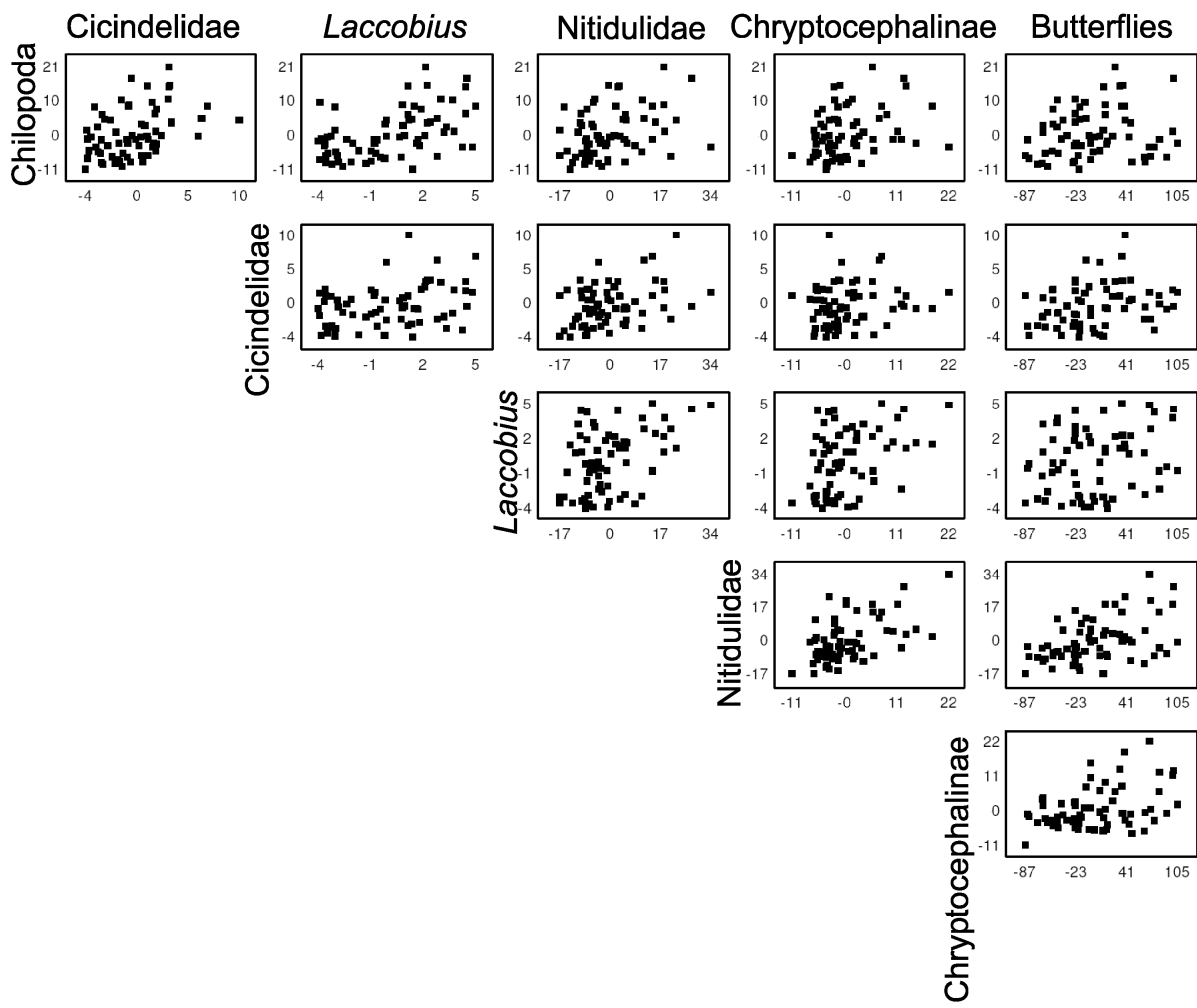

Supplement: Supporting Information S2 — Cross-taxon correlations for species richness (a), species/area ratio (b), and residuals from the species-area relationship (c). (PDF) [file pone.0040018.s002.pdf]
